# Septins From Protists to People

**DOI:** 10.3389/fcell.2021.824850

**Published:** 2022-01-17

**Authors:** Brent Shuman, Michelle Momany

**Affiliations:** Fungal Biology Group and Plant Biology Department, University of Georgia, Athens, GA, United States

**Keywords:** interface, evolution, heteropolymer, septin, domains

## Abstract

Septin GTPases form nonpolar heteropolymers that play important roles in cytokinesis and other cellular processes. The ability to form heteropolymers appears to be critical to many septin functions and to have been a major driver of the high conservation of many septin domains. Septins fall into five orthologous groups. Members of Groups 1–4 interact with each other to form heterooligomers and are known as the “core septins.” Representative core septins are present in all fungi and animals so far examined and show positional orthology with monomer location in the heteropolymer conserved within groups. In contrast, members of Group 5 are not part of canonical heteropolymers and appear to interact only transiently, if at all, with core septins. Group 5 septins have a spotty distribution, having been identified in specific fungi, ciliates, chlorophyte algae, and brown algae. In this review we compare the septins from nine well-studied model organisms that span the tree of life (*Homo sapiens*, *Drosophila melanogaster*, *Schistosoma mansoni*, *Caenorhabditis elegans*, *Saccharomyces cerevisiae*, *Aspergillus nidulans*, *Magnaporthe oryzae*, *Tetrahymena thermophila*, and *Chlamydomonas reinhardtii*). We focus on classification, evolutionary relationships, conserved motifs, interfaces between monomers, and positional orthology within heteropolymers. Understanding the relationships of septins across kingdoms can give new insight into their functions.

## Introduction

**FIGURE 1:**
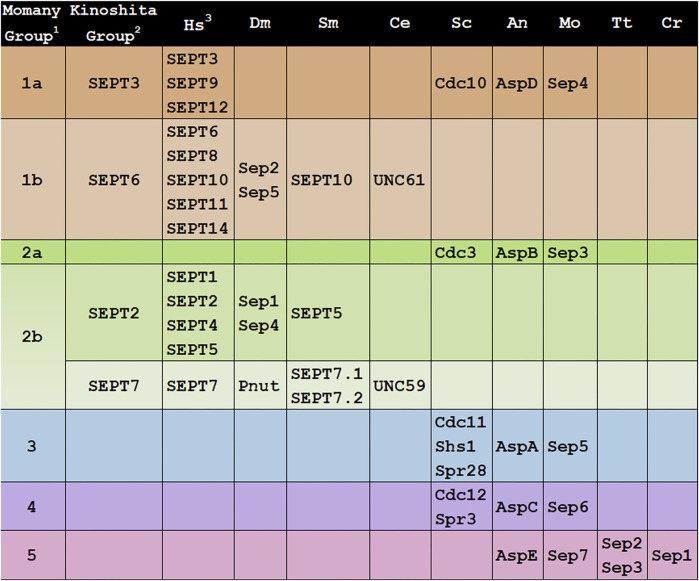
Groupings of septins across Kingdoms. Septin designations for representative model organisms across kingdoms are shown. Protein sequences were retrieved from UniProt, WormBase, NCBI, or FungiDB. ^1^All 161 then-available septin sequences were classified into 5 groups by the Momany lab (Pan et al., 2007). ^2^All 12 then-available human septins were classified into 4 homologous groups, members of which were proposed to substitute for each other in a polymer (“Kinoshita rule”) by the Kinoshita lab (Kinoshita 2003b). ^3^Hs, *Homo sapiens*; SEPT1(*Q8WYJ6*), SEPT2(*Q15019*), SEPT3(*Q9UH03*), SEPT4(*Q6ZU15*), SEPT5(*Q99719*), SEPT6(*Q14141*), SEPT7(*Q16181*), SEPT8(*Q92599*), SEPT9(*Q9UHD8*), SEPT10(*Q9P0V9*), SEPT11(*Q9NVA2*), SEPT12(*Q8IYM1*), SEPT14(*Q6ZU15*); Dm, *Drosophila melanogaster*; Sep1(*P42207*), Sep2(*P54359*), Sep4(*Q0KHR7*), Sep5(*Q7KLG8*), Pnut(*P40797*); Sm, *Schistosoma mansoni*; SEPT5(*KC916723*), SEPT7.1(*KC916724*), SEPT7.2(*KC916725*), SEPT10(*KC916726*); Ce, *Caenorhabditis elegans*; UNC-59 (*CE20165*), UNC-61(*CE47829*); Sc, *Saccharomyces cerevisiae*; Cdc3(*YLR314C*), Cdc10(*YCR002C*), Cdc11(*YJR076C*), Cdc12(*YHR107C*), Shs1(*YDL225W*), Spr3(*YGR059W*), Spr28(*YDR218C*); An, *Aspergillus nidulans*: AspA(*AN4667*), AspB(*AN6688*), AspC(*AN8182*), AspD(*AN1394*), AspE(*AN10595*); Mo, *Magnaporthe oryzae*; Sep3(*MGG_01521*), Sep4(*MGG_06726*), Sep5(*MGG_03087*), Sep6(*MGG_07466*), Sep7(*MGG_02626*); Tt, *Tetrahymena thermophila;* Sep2(*I7M2Q5*), Sep3(*Q240L4*); Cr, *Chlamydomonas reinhardtii*; Sep1(*EDP03113*).

Septin GTPases form nonpolar heteropolymers and are a component of the cytoskeleton (Mostowy and Cossart 2012). The first septins were identified in *Saccharomyces cerevisiae* in the classic cell division cycle (*cdc*) mutant screen. The four prototypical septin mutants, *cdc3*, *cdc10*, *cdc11*, and *cdc12*, made elongated buds and did not complete cytokinesis at restrictive temperature (Hartwell et al., 1970; Hartwell et al., 1973). In the 50 years since their discovery, septins have been identified in organisms across kingdoms and much work has been done to understand septin evolution, structure, assembly, and function (Angelis and Spiliotis 2016; Momany and Talbot 2017; Spiliotis and McMurray 2020; Spiliotis and Nakos 2021; Woods and Gladfelter 2021). The purpose of this review is to allow researchers to understand the relationships of septins across kingdoms so that they can use insights from other systems to inform their own work. We will do this by comparing septins from nine model systems (Figure 1), focusing on classifications based on evolutionary relationships and conserved motifs, including interfaces. We chose these nine organisms because they span the tree of life and are well-studied, but we urge researchers to remember that there is great diversity in septin structure and function and we are covering only a small, though hopefully representative, slice. Much of the knowledge presented in this review builds on many structural and functional studies conducted in many labs over many years. We apologize to our colleagues whose work might be missed.

## Classification and Evolution

The first attempt to classify the fungal septins was in 2001 when the Momany lab analyzed 27 septins from eight fungi (Momany et al., 2001). They concluded that most fungal septins could be grouped with one of the four *S. cerevisiae* prototypical, or core, septins, and referred to each class based on the *S. cerevisiae* septin member (Cdc3, Cdc10, Cdc11, or Cdc12). They also noted a few fungal septins did not appear to group with others and suggested these might be part of a fifth class.

The first attempt to classify the mammalian septins was in 2003 when Makoto Kinoshita placed the 12 human septins known at the time into four groups based on sequence similarity (Kinoshita 2003a). He also proposed that group members were interchangeable within heteropolymers, an idea that is often now called the “Kinoshita rule.” This rule was very important in shaping septin research because while it was clear that septin monomers interacted with each other to form nonpolar heterohexamers and heterooctamers, the principles that governed their assembly were not yet known. These four Kinoshita septin groups were named after their best-studied members: SEPT2 (group also contains SEPT1, SEPT4, and SEPT5), SEPT3 (also contains SEPT9 and SEPT12), SEPT6 (also contains SEPT8, SEPT10, and SEPT11), and SEPT7 (the only group with a single representative) (Figure 1). In 2003 Kinoshita widened his scope with a phylogenetic analysis of 33 septins from two yeasts and three animals (Kinoshita 2003b). He concluded that septins fell into 2-4 groups within these species. He also concluded that there were no orthologs between animal and fungal septins and suggested that this meant lessons learned from septins in one kingdom might not be informative for those in the other kingdom. Contrary to this view and as described below, later analysis of a wider range of genome sequences showed that there was orthology between animal and fungal septins and that some lessons learned could be applied across kingdoms. As more genome sequences became available, other researchers analyzed more septins and found that the Kinoshita classification system worked well for other animals in addition to humans. In work largely focused on mammalian septins, Martinez and Ware (Martinez et al., 2004) renamed the Kinoshita groups “Group I-IV” based on sequence similarity, though that designation doesn’t seem to have been used much since. Later the Yu lab analyzed 78 septins from nine metazoan species, found they all fell into the four Kinoshita groups, and continued to use the Kinoshita Group names to describe them (Cao et al., 2007).

In 2007 the Momany lab performed an extensive phylogenetic analysis including all 161 septin sequences that were publicly available at the time (Pan et al., 2007). They found septins in animals, fungi, and microsporidia (which were later reclassified as fungi), but none in plants or protists. These septins were placed into five classes and were named Groups 1–5 (Figure 1). Animal septins were found only in Momany Groups 1 and 2, but those groups contained subgroups that were generally consistent with the Kinoshita classification (Momany Group 1a = Kinoshita Group SEPT3, 1b = SEPT6, 2b = SEPT2 and SEPT7). The Momany classification Group 2b contained both Kinoshita Group SEPT2 and Kinoshita Group SEPT7 septins.

In the Momany classification, fungal septins were found in all five groups, with Groups 3–5 being unique to fungi (Figure 1). The Group 5 septins were the most different from those in other groups and were only found in filamentous fungi leading the authors to speculate that Group 5 septins either diverged very recently in filamentous fungi or very early with subsequent loss in most species. Interestingly later work showed that the Group 5 septins don’t participate in the heteropolymer like the prototypical core septins (Hernandez-Rodriguez et al., 2014).

As more high-quality genomes became publicly available, septin sequences were identified in organisms outside of animals and fungi including protists, ciliates, chlorophyte algae and brown algae (Merchant et al., 2007; Wloga et al., 2008; Nishihama et al., 2011). In 2011 the Pringle lab proposed that Group 5 septins were ancestral to animals and fungi based on their identification in Alveolata, Heterokonta, and Planta and additional phylogenetic research in fungal septins supports that proposition (Nishihama et al., 2011; Auxier et al., 2019). It is currently unclear if Group 5 septins are a single group or if they might contain multiple groups, a sort of “none of the above” category relative to the core septins in Groups 1–4. An analysis of septins including those from green algae, brown algae, ciliates, and diatoms published in 2013 by the Kawano lab (Yamazaki et al., 2013) suggests that Group 5 fungal septins are a distinct subgroup, separate from septins in green algae and ciliates.

## Heteropolymers

**FIGURE 2:**
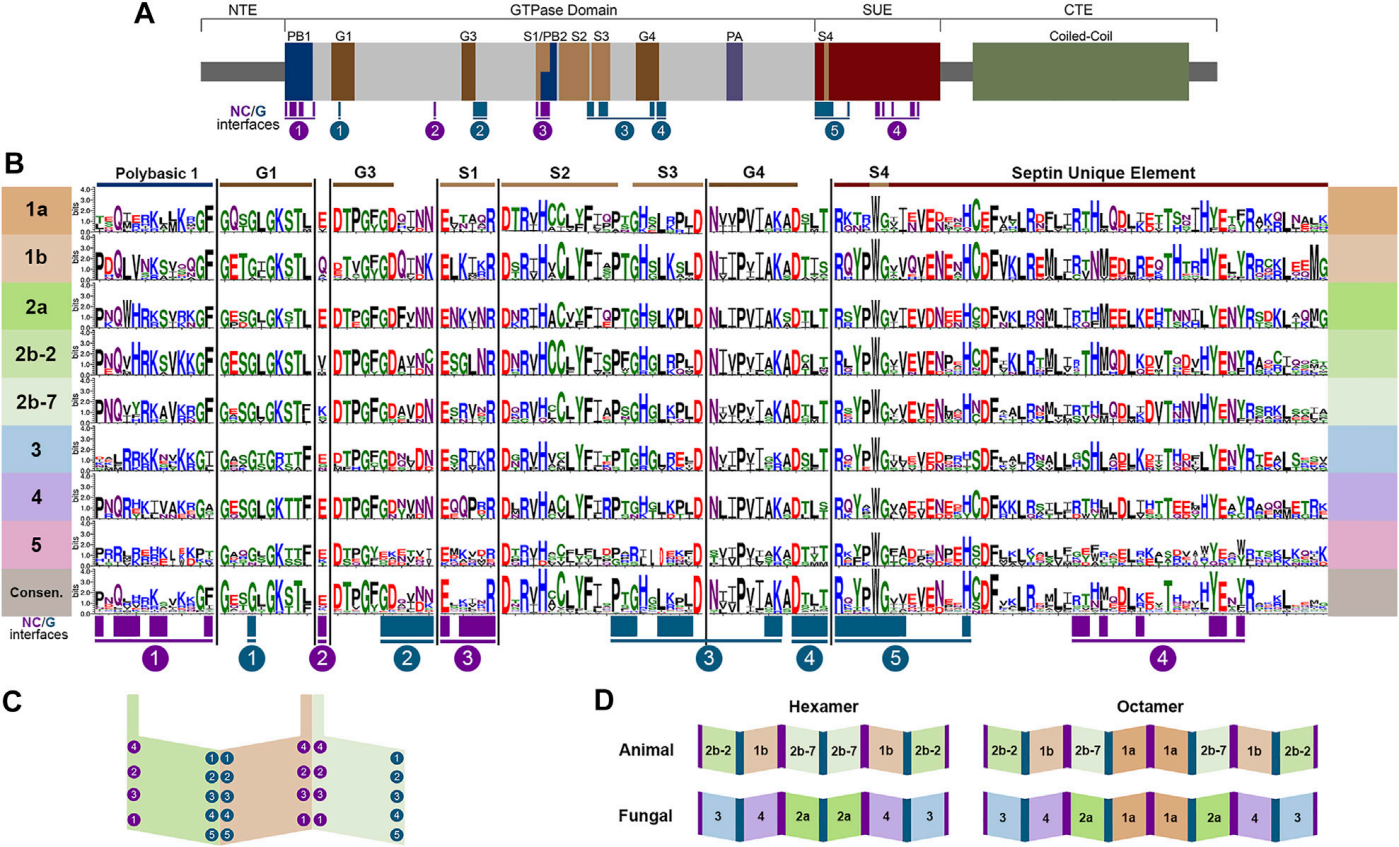
Conserved septin domains and interfaces. **(A)** Septin conserved domains shown to scale modeled on human SEPT7. N-Terminal Extension (NTE), polybasic region (PB), GTPase domain motifs (G1, G3, G4), septin motifs (S1, S2, S3, S4), polyacidic region (PA), septin unique element (SUE), coiled-coil, and C-Terminal Extension (CTE). NC- and G-interface residues shown in magenta and teal. **(B)** Septins were aligned using Clustal Omega (Madeira et al., 2019) and WebLogo 3 (Crooks et al., 2004) was used to highlight conserved regions of septins listed in the table. **(C)** Representation of the SEPT2/6/7 trimer with interacting group designations adapted from Auxier et al. (Auxier et al., 2019). **(D)** Canonical hexamers and octamers from both animals and fungi highlighting positional orthologs.

With the publication of the first septin crystal structure, it became clear that core septin monomers form heteropolymers by interacting with each other via two distinct interface regions, called the G-interface and the NC-interface (Figures 2C,D) (Sirajuddin et al., 2007). Several highly conserved regions first noted in Pan et al. (Pan et al., 2007) were later shown to participate in monomer contacts across interfaces that defined four interacting groups at the NC-interface and five at the G-interface (Auxier et al., 2019; Castro et al., 2020) (Figure 2).

It should be noted that there are far more protein structures available for human septins than for others. Structures of septins from other species would certainly help to clarify many of the outstanding questions in the field. Still, based on known structures and other biochemical and genetic analyses it is clear that canonical octamer and hexamer heteropolymers are assembled following the same general plan; that is to say evolutionarily-related septins occupy the same positions within heteropolymers and bind using the same interfaces (McMurray and Thorner 2019; Mendonca et al., 2019; Soroor et al., 2021). This allows for the comparison of septins across evolutionary groups because they occupy equivalent positions within heteropolymers acting as “positional orthologs,” though it should be noted that positional orthology does not necessarily mean that all activities are equivalent. Since SEPT2 and SEPT7 are within the same subgroup in the Momany classification, but are at different positions within polymers, we show them here as Groups 2b-2 (SEPT2) and 2b-7 (SEPT7). Octamer heteropolymers are formed with a central Group 1a septin dimer, flanked by Group 2 septins (2b-7 in animals or 2a in fungi), flanked by a Group 1b septin in animals or Group 4 septin in fungi, flanked by Group 2b-2 in animals or three in fungi (Figure 2D). The canonical hexamers are identical, except they lack the central Group 1a septin dimer.

Interestingly, *Caenorhaabditis elegans* has only two septins, UNC61 and UNC59. In all classifications, UNC61 was placed with the SEPT6/Group 1b septins. The placement of UNC59 has been less consistent. In the Momany lab analysis UNC59 was in a small Group 2b subclade with two septins from *Caenorhabditis briggsae*. This clade was an outlier, though it was closer to the subclade containing human SEPT2 than to that containing human SEPT7 (Pan et al., 2007). In the Kinoshita and Yu analyses (Kinoshita 2003a; Cao et al., 2007) UNC59 was again something of an outlier but fell into the same clade as human SEPT7.

**FIGURE 3:**
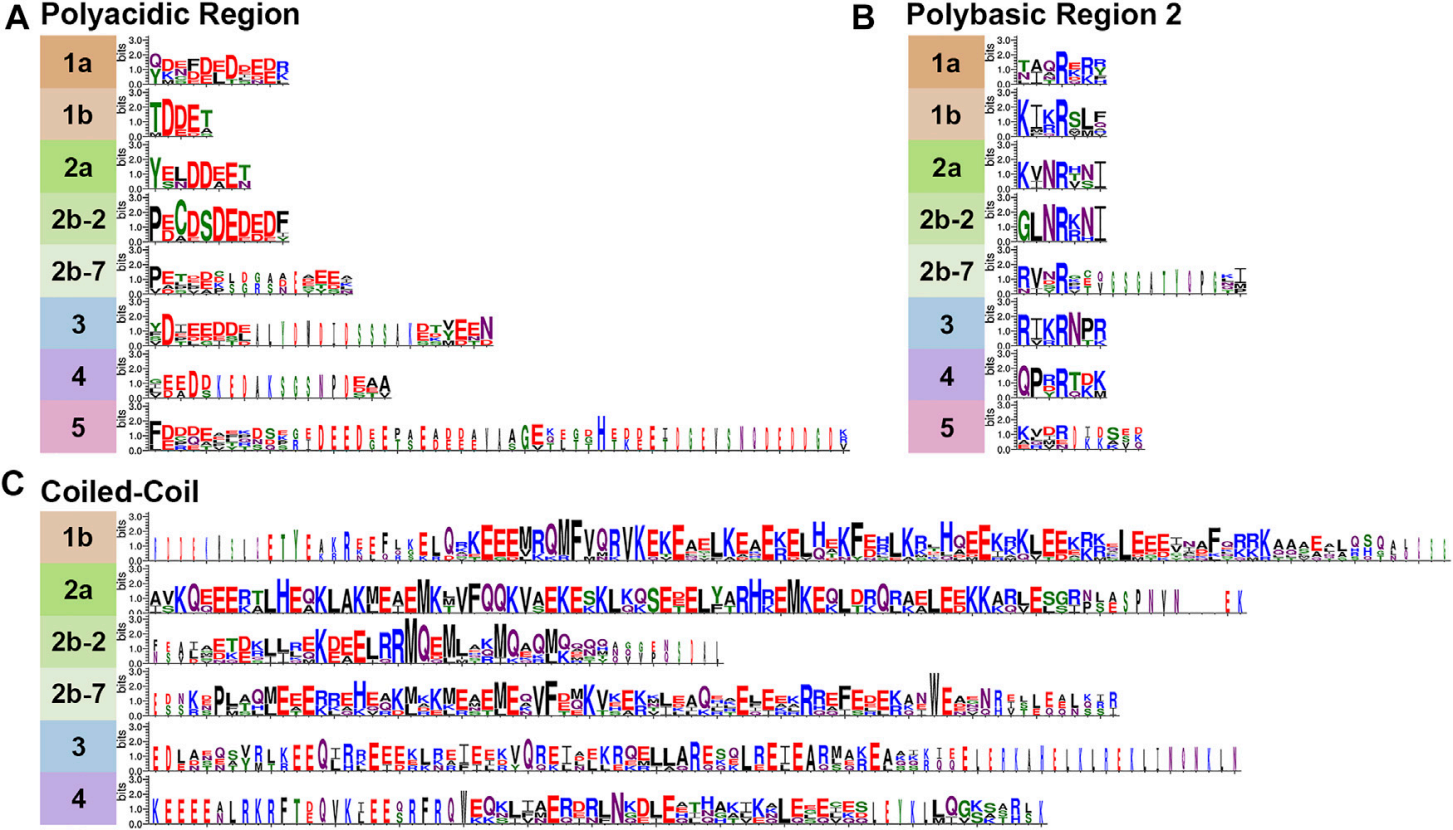
Variable Septin domains. Septins were aligned using Clustal Omega (Madeira et al., 2019) and WebLogo 3 (Crooks et al., 2004) was used to view variable septin domains. **(A)** The polyacidic, **(B)** polybasic 2, and **(C)** coiled-coil regions vary between groups in size and residues.

**FIGURE 4:**
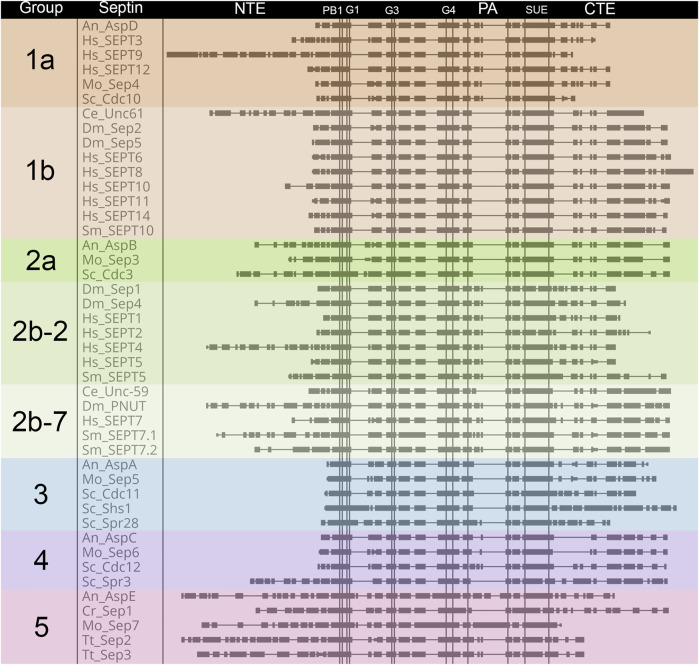
Comparison of septin domain conservation. Large scale view of septins highlighting areas with variable sequence lengths. Septin proteins were aligned using Clustal Omega (Madeira et al., 2019), and placed in alphabetical order within groups. Domains are as shown for Figure 2 and are indicated above the alignment. Vertical lines denote boundaries of domains. Rectangles indicate blocks of conservation. Thin horizontal lines indicate gaps introduced by Clustal to facilitate alignment.

In other model organisms examined here the Group 2b-7 septin is positioned in the interior of the heterohexamer or heterooctamer (Figure 2D). In contrast, UNC-59 appears to be the exposed subunit of the heterotetramer (John et al., 2007). Despite its lack of positional orthology, we show UNC59 as a member of Group 2b-7 in Figures 1–4 because UNC59 was first identified by its homology to the *Drosophila melanogaster* SEPT7 ortholog PNUT (Nguyen et al., 2000). It will be very interesting to see which features of this unusual septin allow it to function as a heterotetramer rather than the more common heterohexamer or heterooctamer.

## Motifs

Not surprisingly based on their evolutionary origin from a common ancestor, septins across species share several well-conserved motifs (Figures 2–4) including those shown in Figure 2A: the polybasic regions (PB1 and PB2), GTPase motifs (G1, G3, G4), septin conserved motifs (S1, S2, S3, S4), polyacidic region (PA), and the septin unique element (SUE) (Zhang et al., 1999; Leipe et al., 2002; Versele et al., 2004; Pan et al., 2007; Valadares et al., 2017; Omrane et al., 2019). Comparing the nine representative model organisms highlights interesting features of the conserved motifs across the five septin groups (Figures 1–4):

### N-Terminal Extension

The length of the NTE varies widely (Figure 4). Interestingly the extended NTE of SEPT9 has been shown to interact with the acidic tails of microtubules and crosslink F-actin, bridging three mammalian cytoskeletal elements (Bai et al., 2013; Smith et al., 2015). This does not appear to be a feature common to other Group 1a septins (even within humans), and may have evolved independently. Among the nine model organisms we examine here, fungal-specific Groups 3 and 4 have very short NTEs with the exception of the *S. cerevisiae* meiotic septin Spr3. Representative Group 5 septins also have longer NTEs than most, but their function is currently unknown.

### Polybasic Regions (PB1 and PB2)

The PBs have been shown to bind phosphoinositides in fungi and humans (Zhang et al., 1999; Casamayor and Snyder 2003; Omrane et al., 2019). PB1 of these model organisms vary in the number of basic residues (between 1 and 7) and their relative positions within the conserved region (Figure 2B). However, none of these septins completely lack basic residues within this N-terminal region. Though less common, acidic residues are present in PB1 of some septins. It has been shown that adding an acidic residue can reduce the ability of PB1 to bind phosphatidylinositides (Casamayor and Snyder 2003).

First identified in 2019 in human septins, PB2 has also been shown to bind phosphoinositides, and in SEPT9 both polybasic regions are thought to increase the selectivity of the septin to particular membrane curvatures (Omrane et al., 2019). PB2 overlaps with the previously identified highly conserved Sep1 (S1) by four residues where it takes part in an NC-interface interacting group (Figure 2). Among the model organisms we analyzed, the presence of a PB2 region is highly conserved across groups; however, except for the highly conserved central arginine residue, the sequence of PB2 is conserved within groups, not across them (Figure 3B).

### GTPase Motifs (G1, G3, G4)

Septins contain three of the five motifs that define P-loop GTPases (Leipe et al., 2002). The G1, G3, and G4 motifs interact with the GTP nucleotide and/or its cofactor. As expected, based on this critical function, the GTPase domains are highly conserved across septins. The Group 5 septins show the most variation in GTPase domains, though the conservation is still high.

### Sep Motifs (S1-S4)

The Sep motifs were identified based on high levels of conservation across multiple species (Pan et al., 2007). Though their function was not clear at the time, it was later found that Sep1, Sep3, and Sep4 were likely involved in contacts between septin monomers within heteropolymers (Auxier et al., 2019). Once more the most variation is seen within Group 5 Sep motifs consistent with data suggesting they do not interact with other septins in the canonical manner (Hernandez-Rodriguez et al., 2014).

### Polyacidic Region

First identified by the Araujo and Garratt labs in 2017 (Valadares et al., 2017), the polyacidic region is thought to interact with PB1 or PB2 at the center of the octamer depending on the conformation of the NC-interface (Castro et al., 2020). As shown for our nine model organisms the septin PA is highly variable in length and acidity across groups, but is conserved within groups (Figures 3A, 4).

### Septin Unique Element

The SUE is a large (53 amino acids) region first identified based on conservation in many septins (Versele et al., 2004). It was later shown to contain contact regions for both NC- and G-interfaces (Auxier et al., 2019). No other functions have been described for this motif.

### C-Terminal Extension

The CTE of septins varies more than the central regions of the protein (Figure 4). One of the CTE features that has drawn notice is the presence or absence of a coiled-coiled. Coiled-coils are important for septin higher-order structure formation, and recent crystal structures of the coiled-coils reveal they interact both within and across polymers (Bertin et al., 2010; Leonardo et al., 2021). Septins from Groups 1a and five do not have predicted coiled-coils (Pan et al., 2007) (Figure 3C). The truncated CTE and lack of a coiled-coil is especially interesting for Group1a septins since they form the central dimer of the heterooctamer (Figure 2D). This central dimer is formed via the NC-interface which in all other heteropolymeric septins is the interface from which the coiled-coils emerge (Figure 2C).

Another feature of note is the presence of an amphipathic helix in many septin CTEs. The distinct hydrophobic and hydrophilic faces of amphipathic helices are thought to allow septins to sense membrane curvature. So far amphipathic helices have been identified in septins from groups 1b, 2b-2, 2b-7, 3, and 4 (Cannon et al., 2019; Woods et al., 2021).

## Conclusion

Septins have duplicated and diversified from their common evolutionary ancestor to perform an amazing array of important functions across species from protists to people. The ability to form heteropolymers appears to be critical to many of those functions and to have been a major driver of the high conservation of many critical domains. Because of this, comparisons can be made between animal and fungal septins that are positional orthologs, though more structural studies on septins from other species are certainly needed to complement the studies on human septins.

The significance of the divergence in polymer composition is still unknown. Perhaps the most striking example of this divergence is in the Group 5 septins which are present in some fungi and protists, absent in animals, and which appear to interact with core septins only transiently. More studies on Group 5 septins from fungi and their non-opisthokont relatives may prove critical to understanding the origins of septins and their ability to interact with each other, with other proteins, and with the membrane. Studying positional orthologs across kingdoms should reveal the history of how septins have been added or removed from polymers and possible conserved functions of septins with shared evolutionary history.
